# Supplementary material for: Antibacterial potency of type VI amidase effector toxins is dependent on substrate topology and cellular context
Source: eLife. 2022 Jun 28;11:e79796. doi: 10.7554/eLife.79796 (PMC9270033; doi:10.7554/eLife.79796)
Supplement: Figure 3—source data 1. [file elife-79796-fig3-data1.pptx]

## Slide 1
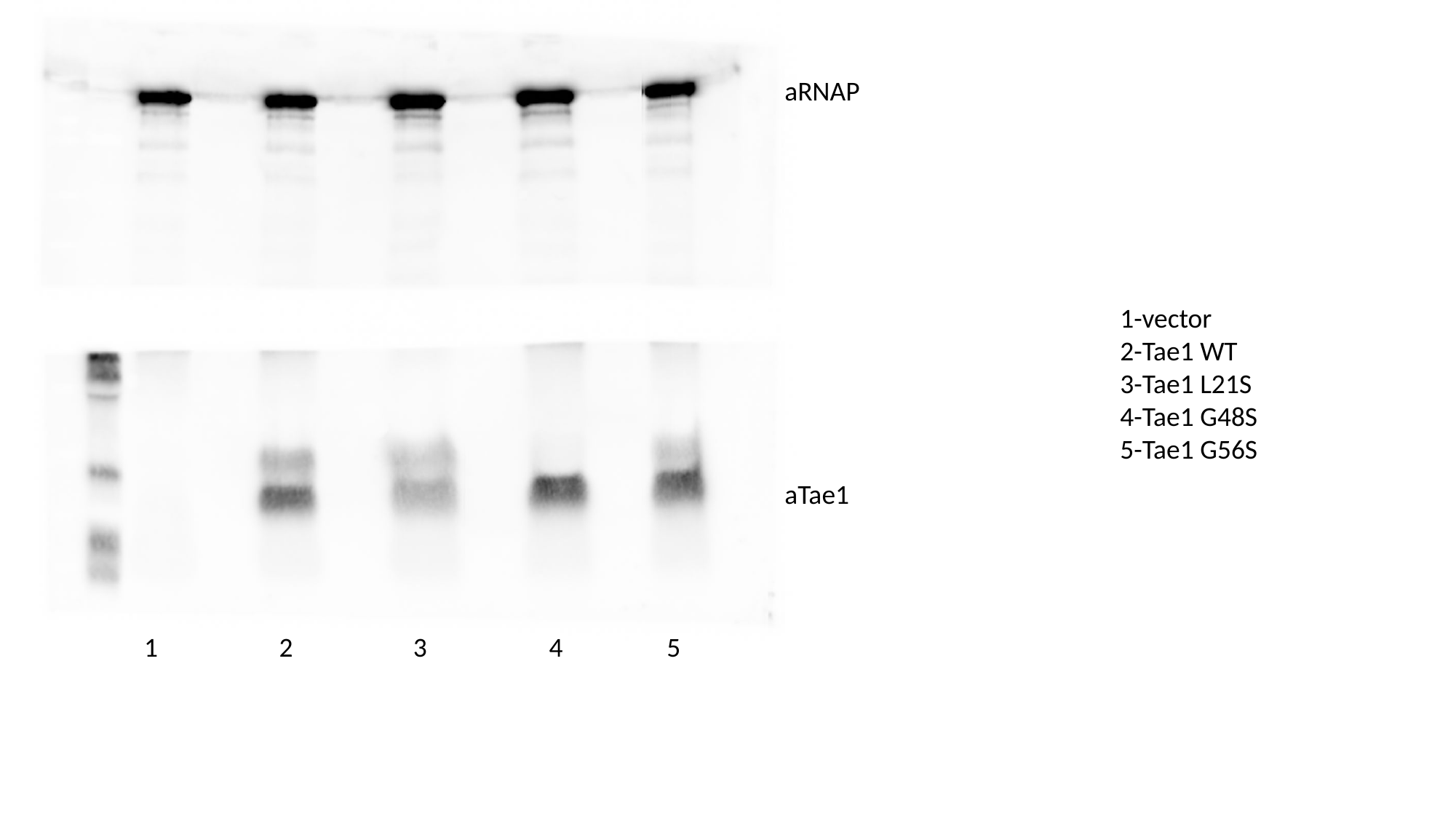

aRNAP
1-vector
2-Tae1 WT
3-Tae1 L21S
4-Tae1 G48S
5-Tae1 G56S
aTae1
1
2
3
4
5

## Slide 2
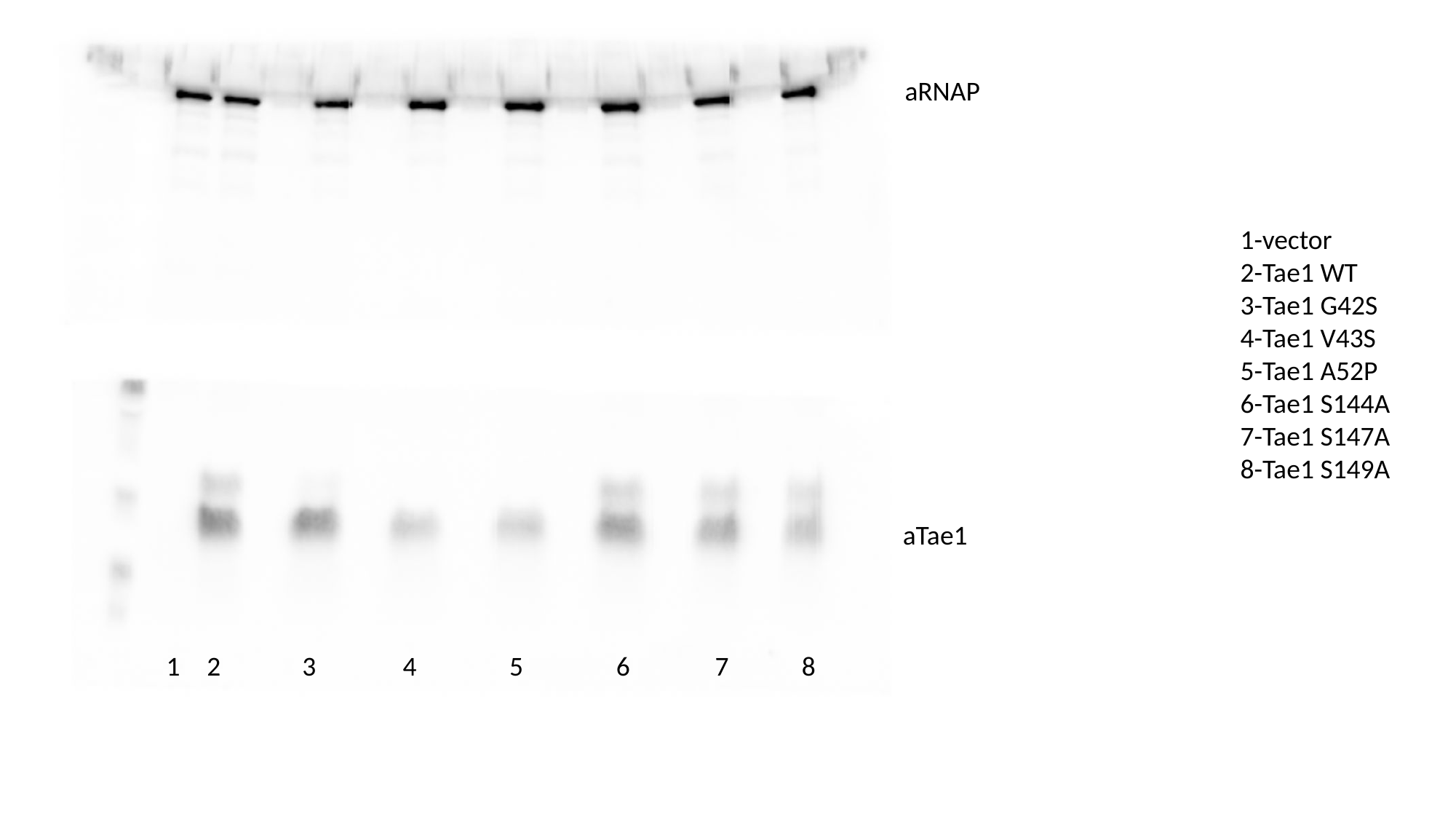

aRNAP
1-vector
2-Tae1 WT
3-Tae1 G42S
4-Tae1 V43S
5-Tae1 A52P
6-Tae1 S144A
7-Tae1 S147A
8-Tae1 S149A
aTae1
1
2
3
4
5
6
7
8
