# Supplementary material for: Antibacterial potency of type VI amidase effector toxins is dependent on substrate topology and cellular context
Source: eLife. 2022 Jun 28;11:e79796. doi: 10.7554/eLife.79796 (PMC9270033; doi:10.7554/eLife.79796)
Supplement: Figure 4—source data 1. [file elife-79796-fig4-data1.pptx]

## Slide 1
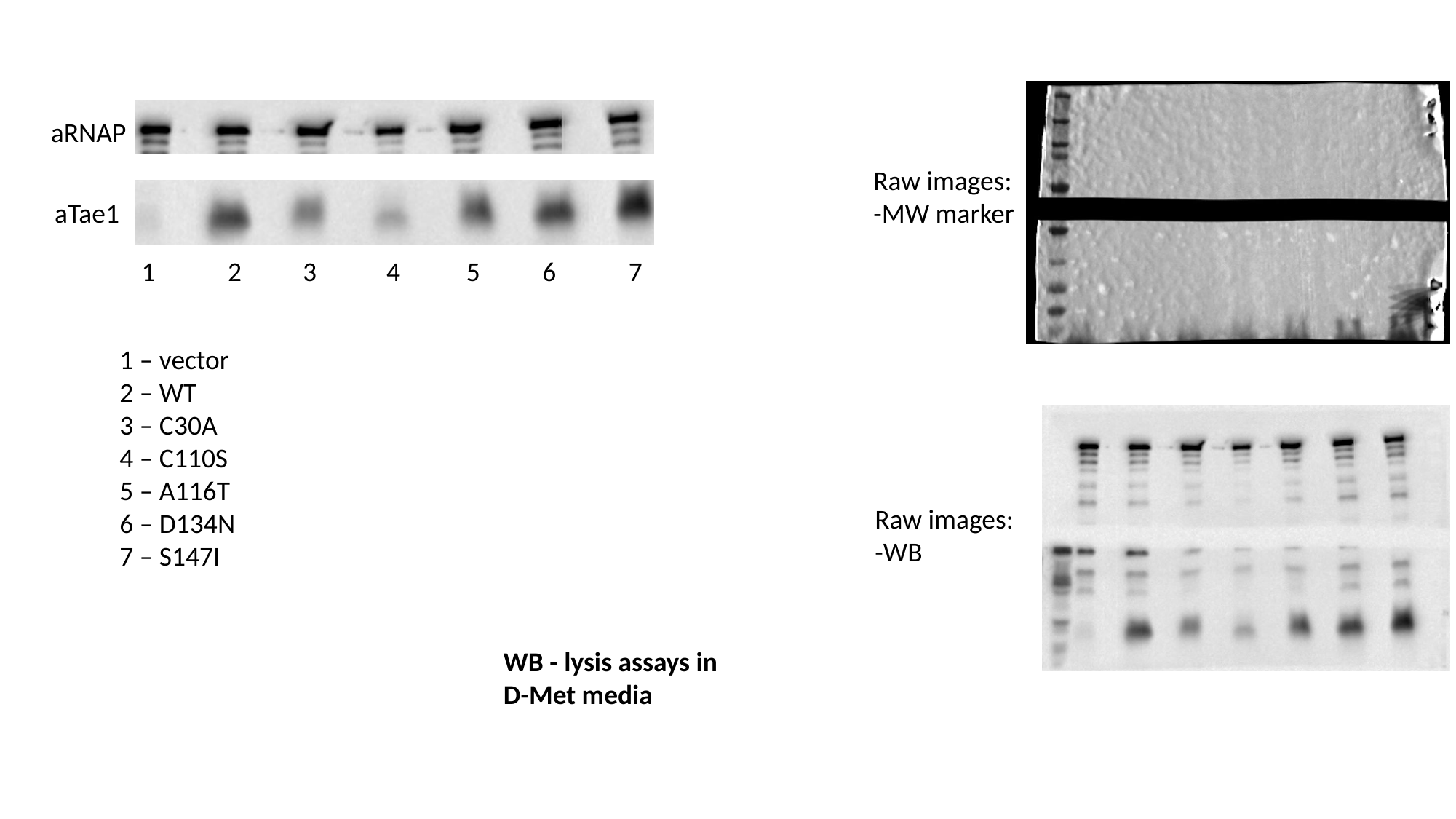

aRNAP
Raw images:
-MW marker
aTae1
1
2
3
4
5
6
7
1 – vector
2 – WT
3 – C30A
4 – C110S
5 – A116T
6 – D134N
7 – S147I
Raw images:
-WB
WB - lysis assays in
D-Met media

## Slide 2
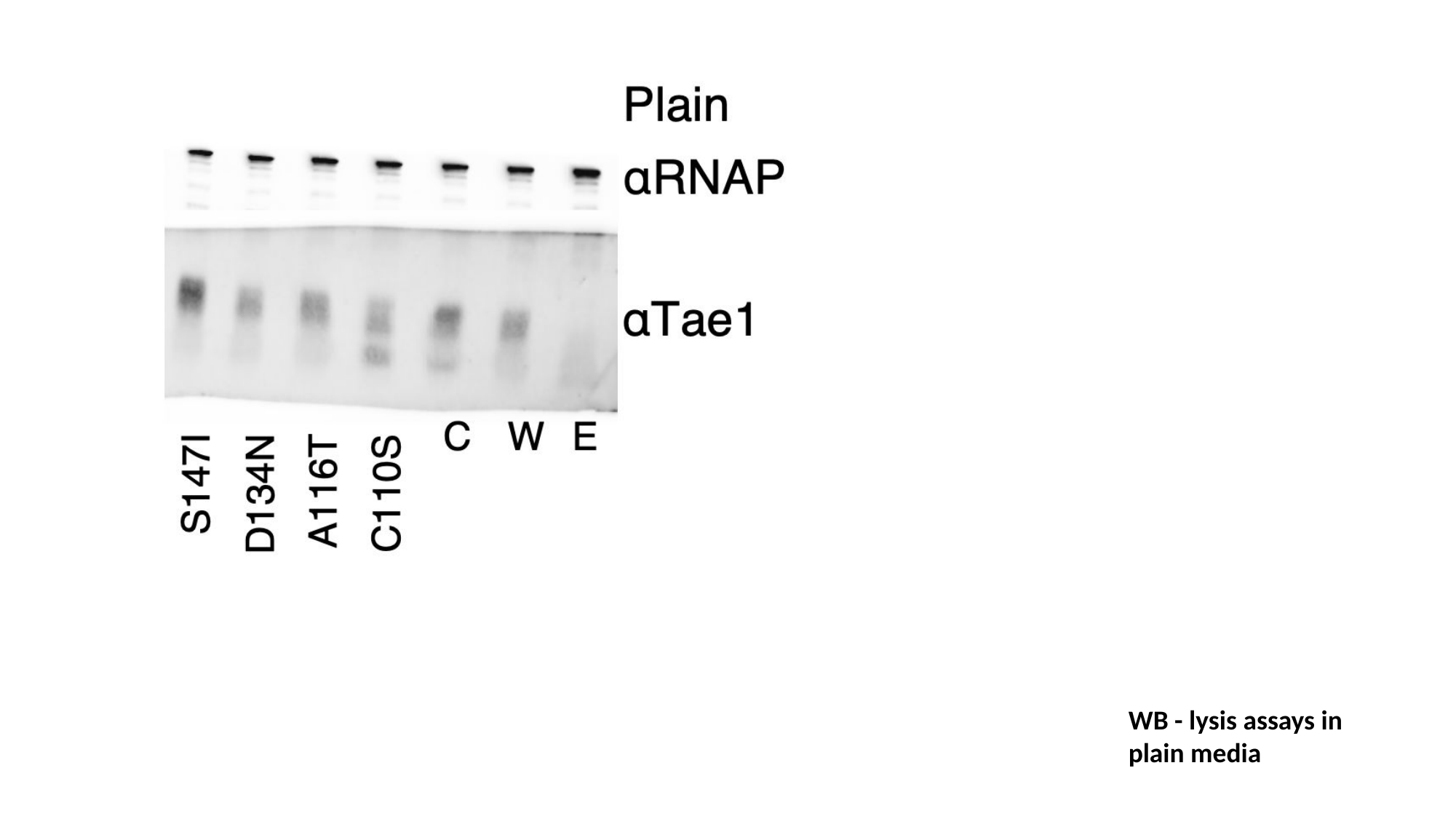

WB - lysis assays in
plain media
