# Supplementary material for: Antibacterial potency of type VI amidase effector toxins is dependent on substrate topology and cellular context
Source: eLife. 2022 Jun 28;11:e79796. doi: 10.7554/eLife.79796 (PMC9270033; doi:10.7554/eLife.79796)
Supplement: Figure 4—figure supplement 2—source data 1. [file elife-79796-fig4-figsupp2-data1.pptx]

## Slide 1
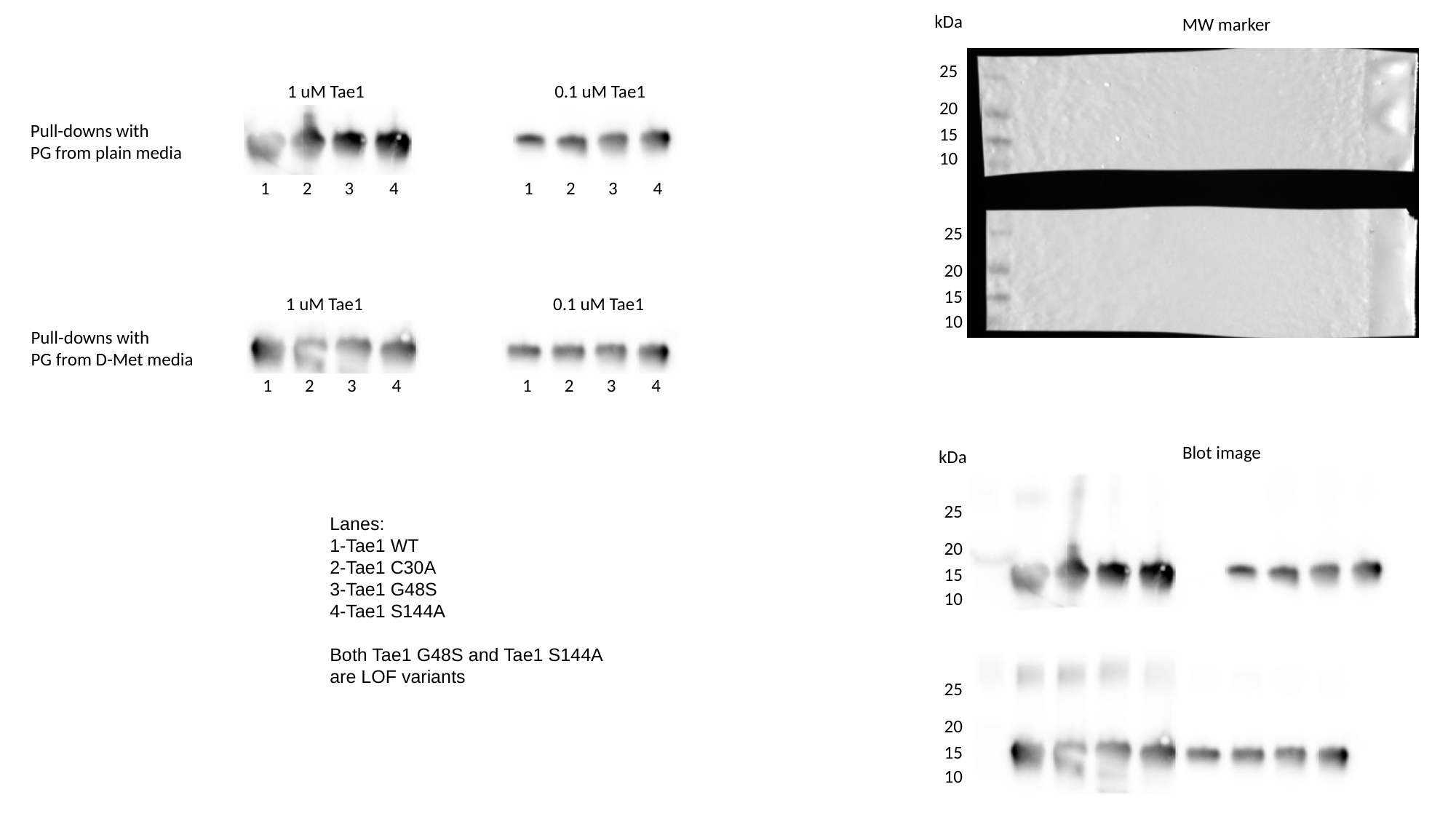

kDa
MW marker
25
1 uM Tae1
0.1 uM Tae1
20
Pull-downs with
PG from plain media
15
10
1
2
3
4
1
2
3
4
25
20
15
0.1 uM Tae1
1 uM Tae1
10
Pull-downs with
PG from D-Met media
1
2
3
4
1
2
3
4
Blot image
kDa
25
Lanes:
1-Tae1 WT
2-Tae1 C30A
3-Tae1 G48S
4-Tae1 S144A
Both Tae1 G48S and Tae1 S144A
are LOF variants
20
15
10
25
20
15
10

## Slide 2
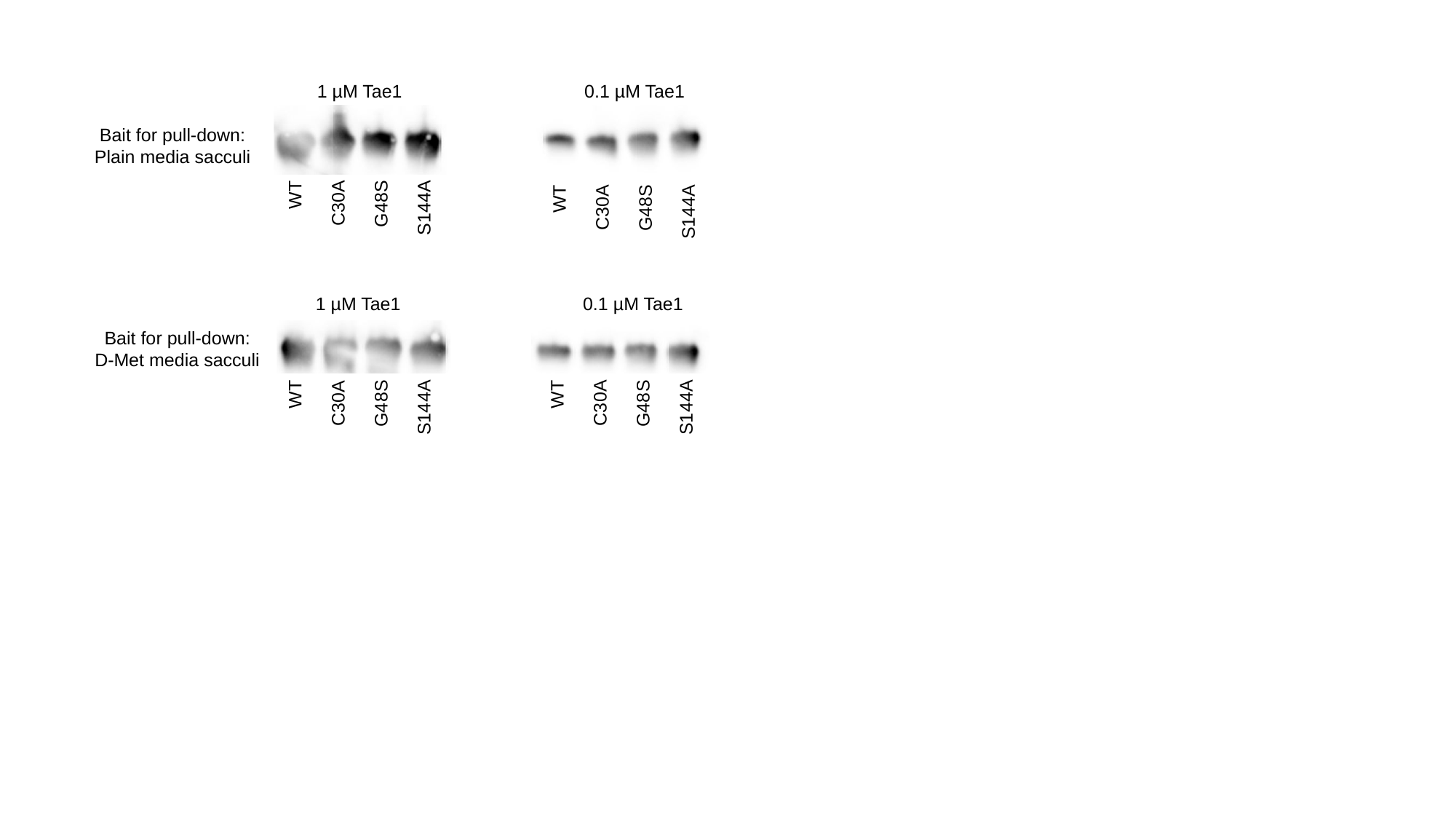

1 µM Tae1
0.1 µM Tae1
Bait for pull-down:
Plain media sacculi
WT
WT
C30A
G48S
C30A
G48S
S144A
S144A
0.1 µM Tae1
1 µM Tae1
Bait for pull-down:
D-Met media sacculi
WT
WT
C30A
C30A
G48S
G48S
S144A
S144A
