# Supplementary material for: Antibacterial potency of type VI amidase effector toxins is dependent on substrate topology and cellular context
Source: eLife. 2022 Jun 28;11:e79796. doi: 10.7554/eLife.79796 (PMC9270033; doi:10.7554/eLife.79796)
Supplement: Figure 6—source data 1. [file elife-79796-fig6-data1.pptx]

## Slide 1
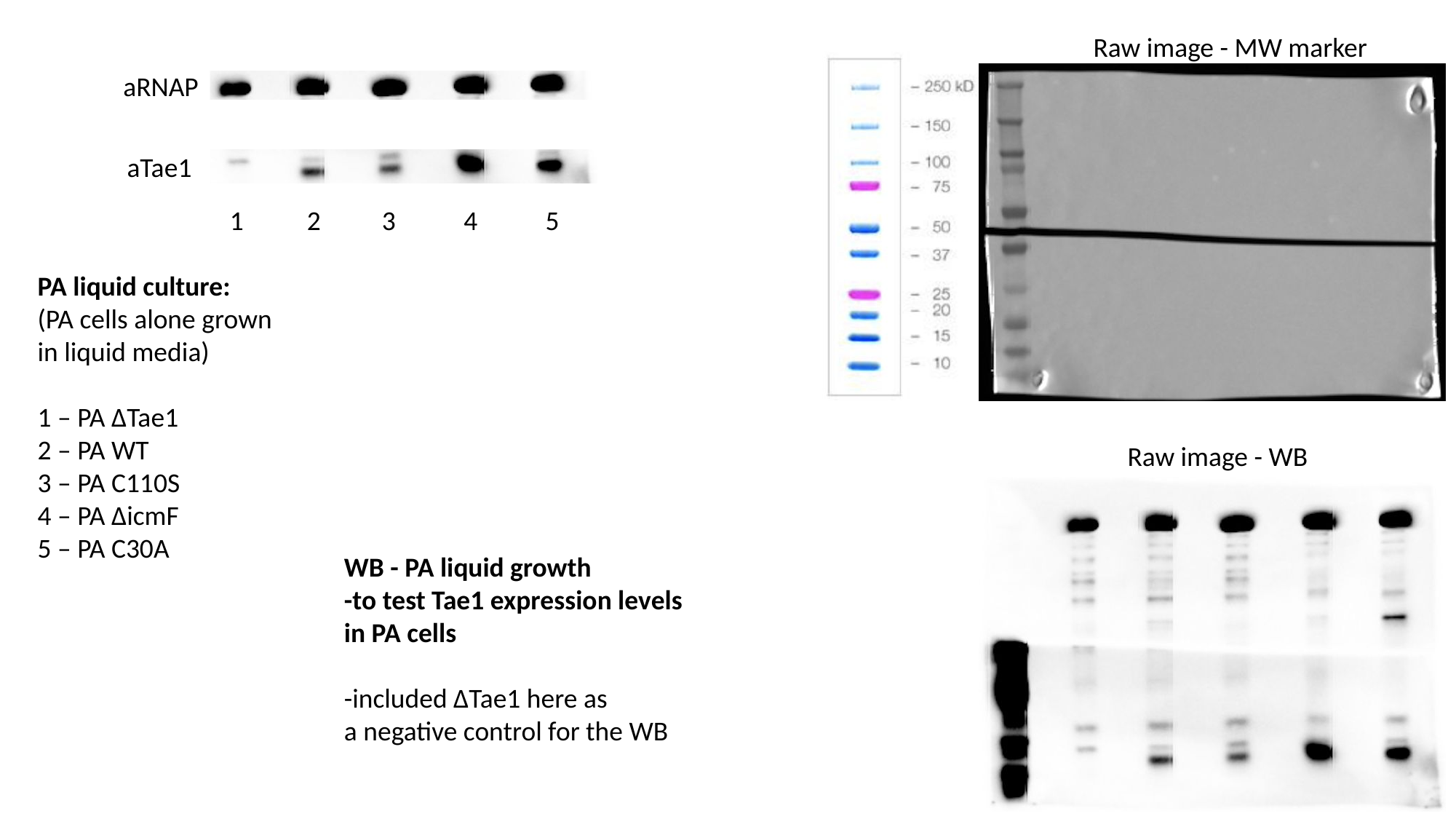

Raw image - MW marker
aRNAP
aTae1
1
2
3
4
5
PA liquid culture:
(PA cells alone grown
in liquid media)
1 – PA ∆Tae1
2 – PA WT
3 – PA C110S
4 – PA ∆icmF
5 – PA C30A
Raw image - WB
WB - PA liquid growth
-to test Tae1 expression levels
in PA cells
-included ∆Tae1 here as
a negative control for the WB

## Slide 2
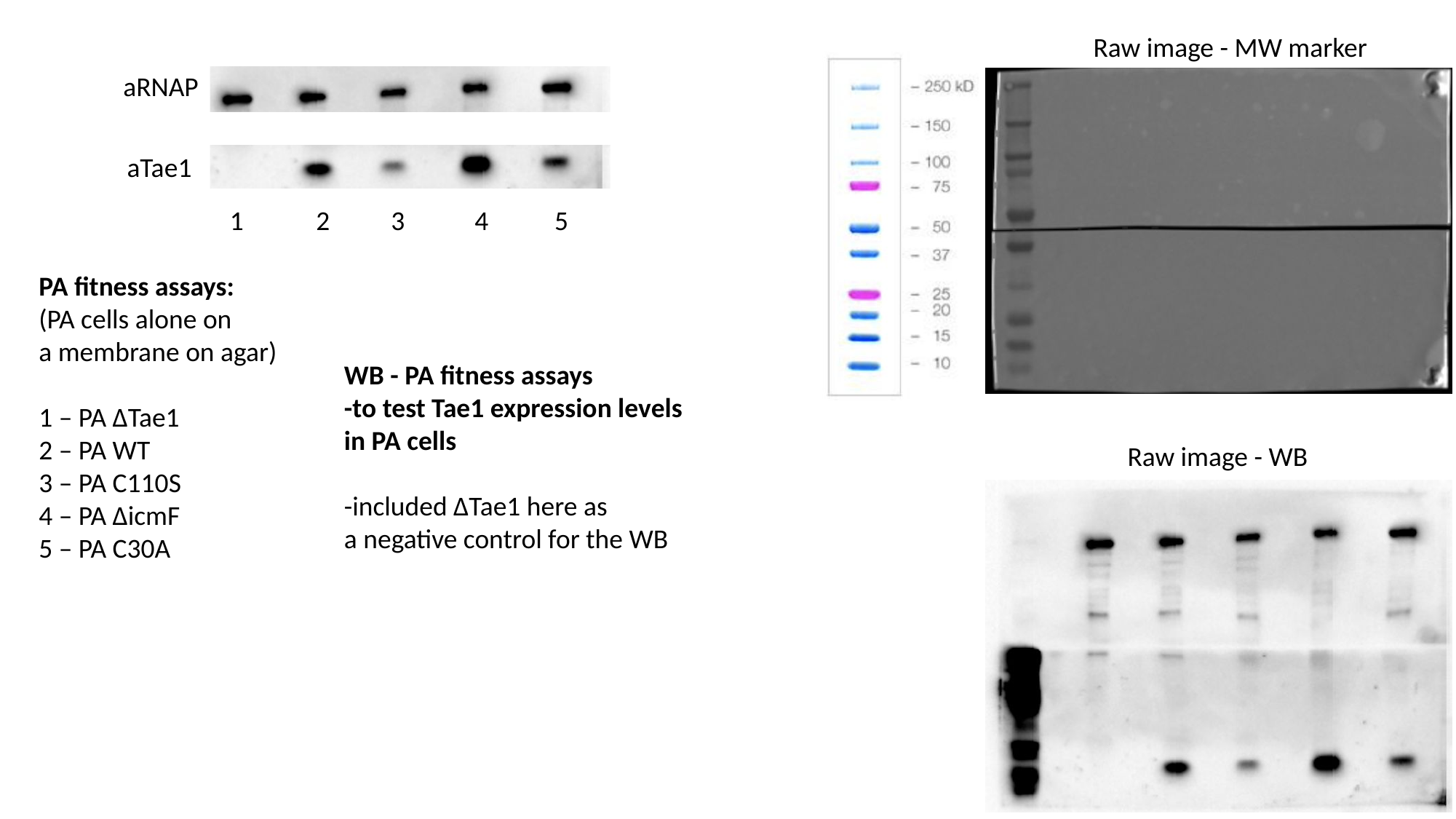

Raw image - MW marker
aRNAP
aTae1
1
2
3
4
5
PA fitness assays:
(PA cells alone on
a membrane on agar)
1 – PA ∆Tae1
2 – PA WT
3 – PA C110S
4 – PA ∆icmF
5 – PA C30A
WB - PA fitness assays
-to test Tae1 expression levels
in PA cells
-included ∆Tae1 here as
a negative control for the WB
Raw image - WB

## Slide 3
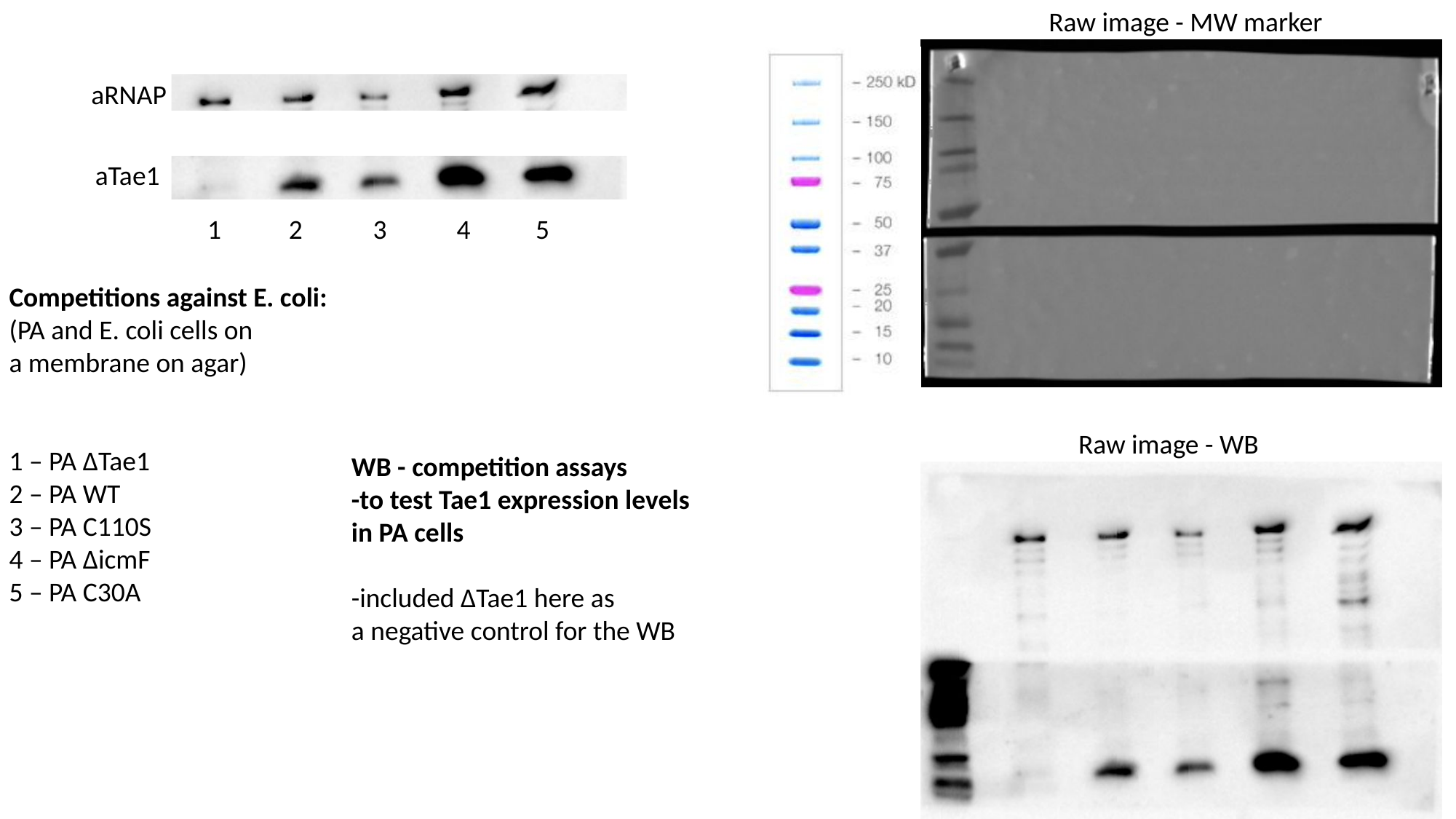

Raw image - MW marker
aRNAP
aTae1
1
2
3
4
5
Competitions against E. coli:
(PA and E. coli cells on
a membrane on agar)
1 – PA ∆Tae1
2 – PA WT
3 – PA C110S
4 – PA ∆icmF
5 – PA C30A
Raw image - WB
WB - competition assays
-to test Tae1 expression levels
in PA cells
-included ∆Tae1 here as
a negative control for the WB
